# Two Decades of 4D-QSAR: A Dying Art or Staging a Comeback?

**DOI:** 10.3390/ijms22105212

**Published:** 2021-05-14

**Authors:** Andrzej Bak

**Affiliations:** Department of Chemistry, University of Silesia, 40007 Katowice, Poland; andrzej.bak@us.edu.pl; Tel.: +48-16-359-11-97

**Keywords:** 4D-QSAR, structure-based SAR, receptor-dependent models, 4D-derived descriptors

## Abstract

A key question confronting computational chemists concerns the preferable ligand geometry that fits complementarily into the receptor pocket. Typically, the postulated ‘bioactive’ 3D ligand conformation is constructed as a ‘sophisticated guess’ (unnecessarily geometry-optimized) mirroring the pharmacophore hypothesis—sometimes based on an erroneous prerequisite. Hence, 4D-QSAR scheme and its ‘dialects’ have been practically implemented as higher level of model abstraction that allows the examination of the multiple molecular conformation, orientation and protonation representation, respectively. Nearly a quarter of a century has passed since the eminent work of Hopfinger appeared on the stage; therefore the natural question occurs whether 4D-QSAR approach is still appealing to the scientific community? With no intention to be comprehensive, a review of the current state of art in the field of receptor-independent (RI) and receptor-dependent (RD) 4D-QSAR methodology is provided with a brief examination of the ‘mainstream’ algorithms. In fact, a myriad of 4D-QSAR methods have been implemented and applied practically for a diverse range of molecules. It seems that, 4D-QSAR approach has been experiencing a promising renaissance of interests that might be fuelled by the rising power of the graphics processing unit (GPU) clusters applied to full-atom MD-based simulations of the protein-ligand complexes.

## 1. Introduction

Nearly a quarter of a century has passed since the eminent work of Hopfinger appeared on the stage [1]; therefore a natural question arises: Is the 4D-QSAR approach still attractive to computational chemists? A rational production/prediction of ADMET-tailored properties (in other words finding a ‘sweet spot’) in the hit→lead→seed→drug cascade is a challenging object of interest for contemporary chemistry, that necessitates at least four German G’s: Glück (luck), Geld (money), Geschick (skill) and Geduld (patience)—the rank order of which depends on the discovery project under scrutiny [2,3]. The intuitive decision-making process of the potential drug nomination or ‘false positives’ eradication at the pre-synthetic stage can be supported by computer-assisted molecular design (CAMD) to minimize the probability of drugs late-attrition according to ‘fail-early fail cheaply’ concept [4]. In fact, a variety of in-silico methods has been proposed in order to transform the compound topology/topography encoded within the symbolic/numeric representations into the property-based chemical space [5]. The methodical observation of structural modifications and the corresponding response variations (e.g., biological activity) for a congeneric series of molecules is fundamental for the multidimensional (mD) quantitative structure-activity relationship (QSAR) modeling—the analysis of analogy and/or similarity is a ‘gold standard’ in the computational chemistry [6]. Moving from the intricate biological systems to physically unrealistic SAR-mediated models on the path ‘towards the prediction paradise’ is usually a backbreaking task more regarded as ‘a triumph of hope over experience’, especially for non-congeneric compounds [7]. As is known, the complex nature of inter/intramolecular interactions engaged in the process of the host-guest formation makes the optimization of the pharmacological response a resource/knowledge CPU-intense issue. Basically, the quantitative mapping of the empirical/theoretical properties/descriptors into the ADMET-friendly molecular potency can be dichotomized into ‘indirect’ (ligand-based) and ‘direct’ (structure-based) procedures, respectively [8]. Conceptually, the receptor-independent (RI) approach stems loosely from the similarity principle, where steric/electronic/lipophilic-alike interchangeable substituents are bound to exert a similar impact on the pharmacological profile (neighbor behaviors) [9,10]. In practice, the ‘reverse image’ of the hypothetical target binding geometry is generated for the ensemble of structurally-related (bio)molecules in the form of pharmacophoric pattern [11,12]. In other words, the pseudoreceptor mapping can specify a spatial (3D) distribution of molecular features that are necessary, but not sufficient for biological activity. As a matter of fact, a number of 3D-QSAR procedures have been practically implemented in the field of medicinal/computational chemistry using molecular interaction/energy field (e.g., CoMFA), molecular surface/volume (e.g., CoMSA) descriptors, respectively [13,14]. Comparative molecular field analysis (CoMFA) is a widespread method that allows to model the influence of molecular shape on steric (Lennard–Jones) and electrostatic (Coulomb) effects engaged in the non-covalent ligand-receptor interactions. Roughly speaking, CoMFA assumes that differences in binding affinities or biological activity for a set of structurally-related compounds can be explained by the comparative investigation of 3D patterns produced within the cubic mesh of points, which encompasses aligned molecules using the selected probe atoms [15]. A number of alternative CoMFA-like protocols have appeared, e.g., comparative molecular surface analysis (CoMSA) that implemented corrections in the molecular shape description, superimposition rules as well as the predictive model quality [16]. The fuzzification of the molecular shape representation with the compound alignment improvement seems to be of special interest owing to a potentially preferable characterization of the binding affinity to the corresponding receptor structure. Hence, CoMSA replaces potential values calculated at single points by the mean potential values specified for surface sectors [17]. Obviously, the rough quantitative comparison of the field-based and surface-related descriptors can provide more realistic picture of the ligand-target recognition scenario; however a question about the underlying biological reality remains still unanswered. From the perspective of ‘drug hunters’ the incorporation of the receptor geometry seem reasonable since (bio)effector multifaceted interaction mode (in Latin ‘ligare’ means ‘to tie’) is mediated by the corresponding spatial arrangement of target atoms in the active interface [18,19]. The qualitative and/or quantitative rationalization of the drug-target binding forces in the receptor-dependent (RD) procedures can be partially achieved using the site-directed molecular docking approach; however the system binding evaluation is still questionable due to deficiency of truly selective scoring functions [20]. Regrettably, there is no a priori ‘rule of thumb’ in a successful voyage or exploration of ADMET-friendly property space; therefore the collaborative fusion of RI and RD procedures is advisable in order to support the chemists’ intuition [21].

**Figure 1:**
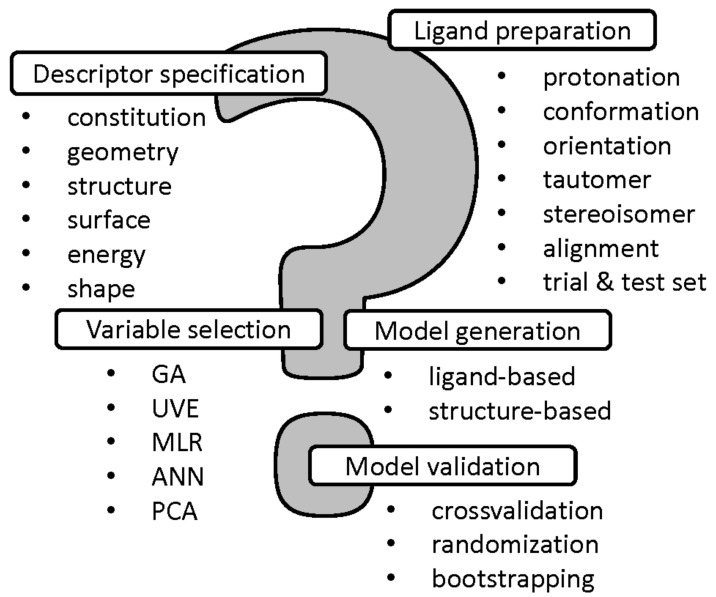
The most common issues in multi-stage QSARs.

In the conventional QSAR methodology a molecular structure is encoded using a pool of descriptors, usually representing a single instance with the fixed conformation, protonation, stereoconfiguration, tautomeric form, etc. Obviously, a molecule is a dynamic object, that can simultaneously change the form of existence or even might exist in many forms at the same time in equilibrium—it is especially important for modeling the biological response profile and/or receptor interaction modes since approximately 50% of marketed drugs are chiral and 25% possess several tautomers, respectively [22]. Hence, the proper ligand preparation and descriptor-weighted specification is valid at the early stages of QSAR modeling with the employment of different protonation states and tautomeric forms [23]. Unfortunately, the alignment problem is another ‘Achilles heel’ of SAR approach, especially for conformationally flexible systems. In order to broaden the applicability of QSAR modeling a range of superimposition-free methodologies have been proposed (e.g., CoMSiA, CoMMA, WHIM) for non-congeneric series of compounds [24]. Common problems of the multi-step QSAR modeling with the variable selection/elimination, model generation and validation are introduced in Figure 1. 

**Figure 2:**
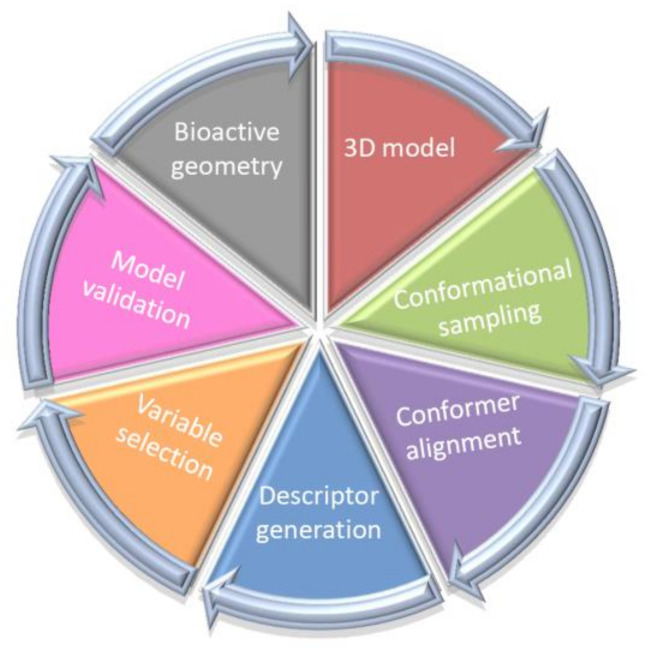
Multi-step 4D-QSAR procedure.

Typically, the postulated ‘bioactive’ 3D ligand conformation is constructed as a ‘sophisticated guess’ (not necessarily geometry-optimized ones) mirroring the pharmacophore hypothesis—sometimes based on an erroneous prerequisite. Unfortunately, the application of alignment-independent descriptors does not address the issues of the proper conformer selection; therefore 4D-QSAR scheme and its ‘dialects’ have been practically implemented as higher level of a model abstraction that allows the investigation of the multiple molecular conformation, orientation and protonation representation, respectively [25,26,27,28]. As a matter of fact, 4D-QSAR methodology diffused quickly into medicinal and computational chemistry becoming a long-established in silico milestone in the field of the computer-aided molecular design. A working definition of 4D-QSAR approach is ‘a set of procedures which allows the construction of optimized dynamic spatial QSAR models, in the form of 3D pharmacophores, which are dependent on conformation, alignment, and pharmacophore-grouping’ [29,30]. In other words, 4D-QSAR can be regarded as a variant of molecular similarity estimation in the Molecular Shape Analysis (MSA), where the substitution of the ‘explicit’ atom-based compound pattern with the ‘implicit’ cube-alike population generates ‘fuzzy’ molecular representation [31,32]. Roughly speaking, 4D-QSAR incorporates some features of the classical mesh-based 3D-QSAR (e.g., CoMFA) as a function of molecular conformation, superimposition and compound fragmentation to produce a molecular shape spectrum (MSS) [33,34]. As a matter of fact, many-fold molecule replications (conformers) allow to increase the chance of mapping the proper receptor form; therefore 4D-QSAR scheme seems to be more probabilistic in nature compared to 3D-QSAR approaches [35,36]. The ‘fuzzification’ of the molecular representation by the cubic occupancy frequency can be optionally augmented using the target data in the receptor-mediated studies [37]. Briefly, the multi-step 4D-QSAR procedure is illustrated in Figure 2.

With no intention to be comprehensive, a review of the current state-of-the-art in the field of receptor-independent and receptor-dependent 4D-QSAR methodology is provided with a brief description of the ‘mainstream’ algorithms. In fact, a myriad of 4D-QSAR methods (namely dialects) have been implemented (grid, neural, lattice, simplex, hybrid, quasar) and applied practically for a diverse range of chemicals as reported so far.

### 4D-QSAR Scientometrics: From Ecstasy to Agony?

Two decades have passed from the moment 4D-QSAR first appeared on the stage; however it is not clear what actually triggers an increasing interest in 4D-QSAR that fades after some years? 

**Figure 3:**
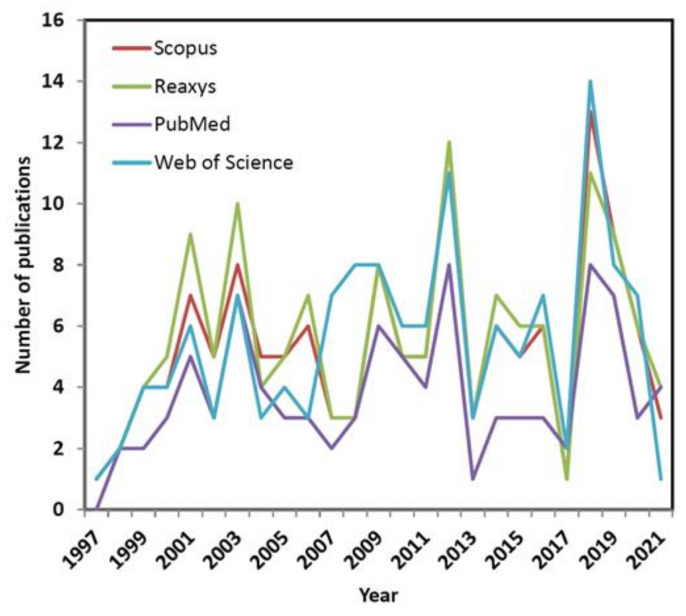
Trends in the number of publications, where ‘4D QSAR’ was queried in the paper’s title, abstract or keyword from 1997 to 2021.

In order to provide the current state-of-the-art an extensive database screening was performed to identify hits, where phrase ‘4D QSAR’ was queried using title, abstract or keywords in the papers published during the last two decades (from 1997 to 2021). In consequence, some puzzling regularities with the fluctuations of interest (namely waves) concerning 4D-QSAR approach were revealed during the exploration of a commercially/freely accessible repositories including Scopus, Reaxys, PubMed and Web of Science databases, respectively. Interestingly, the analysis of database hits in function of time shows an alternating tendency of waxing and waning interest in the conformationally-related QSAR modeling as illustrated in Figure 3.

It seems natural to question what actually induced the growing interest in 4D-QSAR that then diminished after some years? Obviously, the resulting repository data are highly intercorrelated; however noticeable variations in the number of publications per year are observed, probably due to different search protocols implemented in the database engines. On the other hand, some general trends are common for all the investigated databases. Not surprisingly, the initial ‘ecstasy’ is mirrored in the rapid growth of the 4D-related reports at the beginning of new millennium. Unfortunately, in the next decade the slowdown (‘plateaux’) in the number of published papers was observed (with an exception of year 2012) presumably owing to CPU-demanding calculations as well as the reached limits of the ligand-based protocols. The promising renaissance of interests in the receptor-mediated 4D-QSAR has been recorded recently (see Figure 3) that might be fueled by the rising power of the graphics processing unit (GPU) clusters applied to full-atom MD-based simulations of protein-ligand complexes [38].

## 2. 4D-QSAR Dialects: Towards ‘Magic Bullet’

### 2.1. Grid 4D-QSAR Strategy

The conventional cell-based Hopfinger’s 4D-QSAR coding system employs an ensemble of cubic shape-like descriptors that are calculated for the multiple molecule conformational/alignment states as the ‘fourth pseudo-dimension’ [39]. The enhancement of the 3D approaches by considering the additional dimension has been successfully applied in molecular modeling for the search of the active conformation and orientation in binding/active site of the conformationally flexible molecules [40,41,42].

The geometry-optimized molecules are used as the initial structures in order to produce the compound trajectory in the molecular dynamics simulations (MDs) with the generation of the conformational ensemble profile (CEP). The individual conformers are superimposed on the most active (reference) molecule according to the active analogue approach (AAA) hypothesis—the maximum common substructure (MCS) for compounds should be chosen in the superimposition mode. Roughly speaking, the multiple alignment rules offer a possible solution to a system that involves more than one binding mode or a dependence on different regions of the ligand molecule—the alignment issue can be regarded as a search and sample operation corresponding to the conformational profiling [43,44,45,46]. Moreover, molecules can be divided into ‘functional pieces’ called interaction pharmacophore elements (IPEs) that correspond to the types of ligand-receptor interactions [47,48]. Subsequently, a series of molecules is placed in the lattice space and the so-called grid cell occupancy descriptors (GCODs) are calculated using singular cubic cell resolution set to 1, 2 and 0.5 Å, respectively [49,50].

The extension of classical GCODs, namely charge descriptors, were engaged in our calculations with the absolute charge occupancy (A_q_) for the specified IPE of compound *c* calculated as follows:

(1) $$A_{q}\left(c,i,j,k,N\right)=\overset{T}{\underset{t=0}{\sum }}O_{t}\left(c,i,j,k\right)\times q/m$$

where *m* is the number of the atoms of compound *c* that appear in the cell (*i*, *j*, *k*) at time *t*, *q/m* is the mean value of partial atom charges present in some cells at time *t*, *T* is the time in MDs, and *N* is the number of sampling MDs steps.

The joint (J_q_) and self-charge occupancy (S_q_) descriptors with the most active reference compound *R* were specified as:

(2) $$J_{q}\left(c,i,j,k,N\right)=\overset{T}{\underset{t=0}{\sum }}O_{t}\left(c,i,j,k\right)\cap O_{t}\left(R,i,j,k\right)\times q/m$$

(3) $$S_{q}\left(c,R,i,j,k,N\right)=\overset{T}{\underset{t=0}{\sum }}\{O_{t}\left(c,i,j,k\right)-[\sum_{t=0}^{T}O_{t}\left(c,i,j,k\right)\cap O_{t}\left(R,i,j,k\right)]\}\times q/m$$

**Figure 4:**
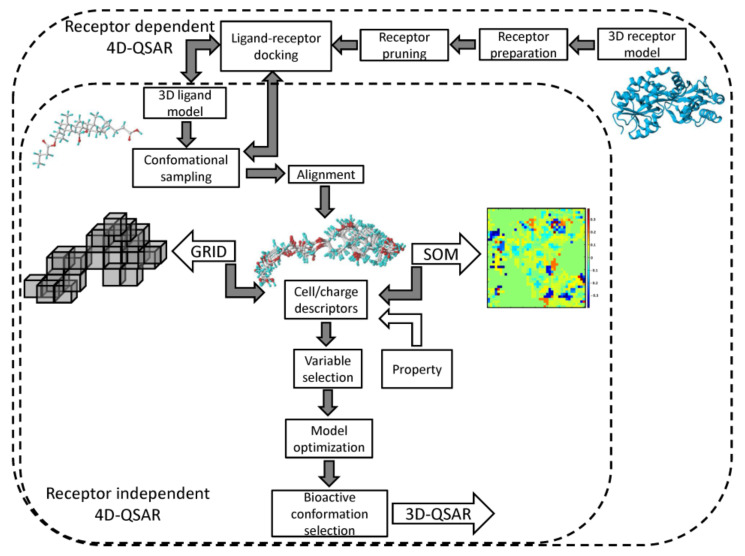
Workflow of receptor dependent (RD) and receptor independent (RI) cell/SOM-based 4D-QSAR strategies.

Each superimposition generates a characteristic grid cell occupancy/charge distribution for a specified molecular trajectory. The grid cells are unfolded into vectors with molecules as objects (rows) and occupancy/charge variables as descriptors (columns). The pool of the cell descriptors can be enhanced by the additional non-GCODs, for instance, lipophilicity coefficients (clogP). The formed array is applied to approach the structure–activity relationship with PLS method conjugated with the variable selection/elimination procedures (e.g., UVE/IVE or GFA) in order to specify the preferred spatial property distribution. In fact, the data reduction is applied to construct the minimal set of similarity/diversity descriptors that approximate the essence of the host-guest interactions [51]. The postulated ‘active conformation’ of each molecule specified on the basis of the extensive sampling of the conformational/alignment freedoms can serve as a ‘preprocessor’ for a subsequent 3D-QSAR analysis (e.g., CoMFA), as shown in Figure 4.

**Figure 5:**
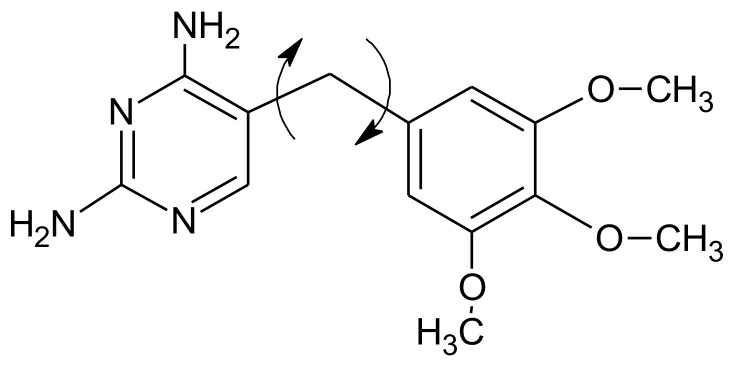
Flexible bonds in trimethoprim.

Grid-based RI-4D-QSAR paradigm was successfully implemented for mapping the 3D-pharmacophore sites of drug targets, for instance, benzodiazepine GABA_A_ receptor, *Mycobacterium tuberculosis* monophosphate kinase (TMPKmt), HIV-1 integrase/protease/ reverse transcriptase, glycogen phosphorylase, dihydrofolate reductase, cytochrome P450, p38-mitogen-activated protein kinase (p38-MAPK) or serotonin transporter (SERT) [40,42,45,46,47,49]. As a first case study, the training set of the substituted 2,4-diamino-5-benzylpyrimidine inhibitors of *E. coli* dihydrofolate reductase (DHFR) was analyzed in 4D-QSAR due to noticeable system conformational flexibility (DHFR-benzylpyrimidine inhibitors have two principal torsion angle degrees of freedom) [1]. As a matter of fact, 4D-QSAR preferred the optimized 3D-QSAR models which include GCODs associated with the ‘fixed’ parts of the structures (see Figure 5)—grid cells near the 2-amino group of the pyrimidine ring were specified as crucial to the compounds inhibitory profile. 

Depending on the alignment rules and conformational analogue flexibility 4D-QSAR identified slight variations among the binding modes of molecules as differences in both the location and/or occupancy values of the GCODs related to ‘constant’ chemical structures. The single ‘active conformation’ was specified as the lowest-energy conformer state which maximizes the predicted activity using the best 3D-QSAR model.

A distinct site-directed 4D-QSAR approach has been promoted recently, where the resultant 3D-pharmacophore pattern is directly dependent upon the explicit geometry of the binding/active pocket in order to capture the potential induced-fit phenomena, especially for the conformationally flexible ligand analogues [52,53,54,55]. Practically, some geometric and force-field restrains are imposed due to distance-dependent short-range characteristics of the host-guest interactions; therefore receptor pruning is advisable to scale down the protein/enzyme to manageable size that embraces the ‘lining’ of the binding/active site [56]. The adopted spatial distribution of the ligand descriptor/property space is mediated by the corresponding mapping of target electronic, steric or lipophilic patterns using the molecular docking procedure [57,58]. The CPU-intense MD calculations (computational time and resource constraints) of the flexible ligand and partially rigid target can provide insight into the potential mechanism of ligand-receptor interactions.

In practice, the cell-based RD-4D-QSAR procedure was applied in modeling of Rho-associated protein kinase inhibitors, HIV-1 protease inhibitors, peptidemimetic inhibitors of *Trypanosoma cruzi* trypanothione reductase (TR) or inhibitors of glycogen phosphorylase (GPb). As a case example, RD-4D-QSAR models were constructed for a series of peptides reversible inhibitors of *Trypanosoma cruzi* trypanothione reductase that was used in the alignment step [56]. The enzyme model was derived from PDB crystal structure and the receptor pruning was performed to limit the time and computational cost of the practical RD-4D-QSAR analysis, respectively. The set of peptide–TR complexes was generated followed the reference ligand bound orientation/conformation (pose) in the active site. Subsequently, the conformational profile recorded from each peptide-TR MD sampling was placed in a reference cell lattice. The GFA-optimized RD models showed to be not only statistically meaningful, but also robust in terms of the external predictivity. The ‘active conformation’ of each peptide-TR complex was hypothesized regarding the model performance and superimposition mode. Moreover, RD 4D-QSAR models also qualitatively ‘captured’ the valid regions of the TR receptor.

### 2.2. Neural 4D-QSAR Methodology

A neural formalism with the engagement of the self–organizing maps (SOMs) for generation of a fuzzy 4D–QSAR–like representation of the conformational space was proposed as an alternative to the classical Hopfinger’s strategy, in namely SOM-4D-QSAR [59,60]. The adaptive and competitive Kohonen algorithm was used in order to produce planar (2D) topographic maps, that represent the signals from chosen atoms of the molecular trajectory (see Figure 4). As a matter of fact, a sphere specified in space by a singular neuron corresponds to a particular unit cube in the conventional 4D-QSAR approach [61]. On the whole, the SOM–4D–QSAR cascade consists of the subsequent operational steps: 

*Step 1*: *Model building*—specification of a spatial geometry for each molecule in the analyzed ensemble. Practically, each of the 3D structures can start the conformational sampling; however the initial geometry optimization is advisable.

*Step 2: Superimposition*—selection of the trial alignment. Basically, the trial superimposition is produced on the most active compound (AAA approach) with FIT procedure to encompass the whole bonding topology in the maximal common structure (MCS).

*Step 3*: *Interaction Pharmacophore Elements* (*IPE*)—compounds are partitioned into subsets of atoms acting a privileged role in the modeled phenomena, e.g., aromatic, hydrogen bond donors, hydrogen bond acceptors, polar positive/negative partial charge, and unrestricted (all) atom type.

*Step 4*: *Conformational Ensemble Profile (CEP)*—the dynamic simulations of molecular system are conducted for sampled conformers that are used in the subsequent comparative analysis. The geometry–optimized models are employed in the initial step in order to produce molecular trajectory and the partial atomic charges using the semi-empirical methods (e.g., PM3 or AM1).

*Step 5*: *Comparative SOM mapping*—the spatial coordinates and partial atomic charges are engaged as the input to form a 2D topographic map. In the course of training these data are distributed among neurons resulting in the sum occupancy or mean charge maps.

*Step 6*: *Variable reduction and model validation*—a SAR relationship is modeled using the PLS algorithm and LOO-CV conjugated with the IVE-PLS procedure for uninformative variable elimination. External model validation is also monitored in order to measure the predictive ability for the external test set. A vast sets of training/test samplings can be monitored by the iterative Stochastic Model Validation (SMV) scheme [62].

Self-organizing RI 4D-QSAR approach was applied to generate a fuzzy ‘cubic-like’ representation of the conformational space for modeling dihydrofolate reductase inhibitors, benzoic acids, azo dyes, steroids, HEPT analogues or the transdermal penetration effect (SKIN) and intestinal absorption enhancement (PAMPA). In order to address the issue of the molecular flexibility the antiviral profile of substituted 1-[2-hydroxyethoxy)methyl]-6-(phenylthio)-thymines (HEPT) analogues was investigated using 4D-QSAR method [60]. Generally, charge descriptors gave better models compared to the occupancy descriptors. Our model also satisfied the ‘butterfly wing’ pattern properly indicating the interactions of the side chains.

The structure-based variant of the SOM-4D-QSAR paradigm has been proposed for modeling of dye-fiber affinity of anthraquinone derivatives [63]. The implemented RD 4D-QSAR approach focused mainly on the ability of mapping dye properties to verify the concept of ‘tinctophore’ in dye chemistry. The neutral (protonated) and anionic (deprotonated) forms of anthraquinone scaffold were examined in order to deal with the uncertainty of the dye ionization state. The results are comparable to both the neutral and anionic dye sets regardless of the occupancy and charge descriptors applied, respectively. It is worth noting that the SOM-4D-QSAR behaves comparably to the cubic counterpart which is observed in each training/test subset specification.

### 2.3. Lattice 4D-QSAR Approach

**Figure 6:**
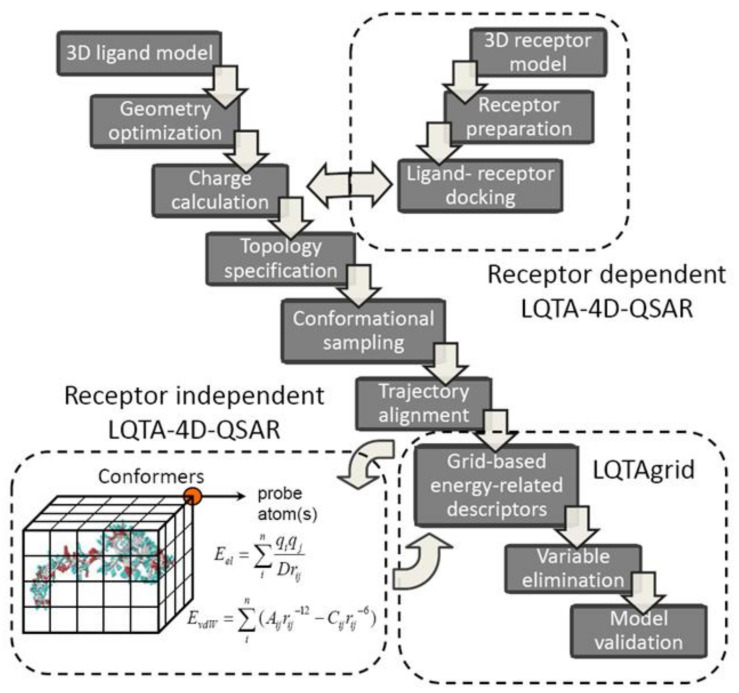
Flowchart of LQTA protocol.

A new protocol, named Laboratorio de Quimiometria Teórica y Aplicada (LQTA), has recently evolved from 3D/4D-QSAR methods. As a matter of fact, the lattice-related (L), charge-based (Q), time-dependent (T) analysis (A) explores jointly the unique features of CoMFA and 4D-QSAR using the intermolecular atom-probe interaction energies (Coulomb and Lennard-Jones) at each grid point of the conformational space sampled in the molecular dynamic simulations [64]. Moreover, an evolution of receptor independent LQTA has been proposed recently implementing the coupled combination of molecular docking and dynamic simulation in order to predict/represent the kinetic state of compounds at target binding site [65]. A simplified flowchart of the RI and RD-LQTA-4D-QSAR methodology is provided in Figure 6. Firstly, the spatial ligand models are optimized using semi-empirical or ab initio methods and the partial atomic charges are calculated at the (semi)quantum modeling level (ChelpG). Hence, the topographic/topologic structural characteristic for each molecule is specified using PRODRG on-line server or Topolbuild software as an input to Gromacs trajectory generator [66]. Typically, the system is initially neutralized, minimized and equilibrated and subsequently 500 ps trajectory space sampling is performed to produce conformational ensemble profile (CEP) considering explicit aqueous medium (SPC/E water model) [67]. The generated MDs frames are arbitrarily superimposed on the reference molecule (ligand-based alignment) by matching the atomic positions—the root-mean-square of the distances (RMSD) between the corresponding atom pairs is monitored. The aligned CEPs are then enclosed in a hypothetical, regular 3D cell box with grid spacing of 1 Å to calculate the energy-based interactions descriptors using LQTAgrid procedure.

The spatial map of the electrostatic (Columbic) and steric (Lennard-Jones) potentials is generated using probe atoms, ions or functional groups (CH_3_^+^, H_2_O, CO_2_^−^, NH_3_^+^, etc.) at the evenly distributed virtual lattice of points, where 3D-energy interaction descriptors (IEDs) are computed at each cubic intersection according to formulas mentioned elsewhere [68]. The multileveled data reduction procedure (digital filter) as a preprocessing (block scaling) is then applied on the separated array of electrostatic and steric potentials with a priori elimination of distant (cutoff = 30 kcal/mol), poorly distributed (variance lower than 0.01 kcal/mol) and correlated (|R| < 0.3 or 0.2) mesh descriptors:

(4) $$\;\;E_{st\left(x,y,z\right)}\;or\;E_{el\left(x,y,z\right)}\geq 30\;\to \;E_{st\left(x,y,z\right)}\;or\;E_{el\left(x,y,z\right)}=30+\log\left(E_{st\left(x,y,z\right)}\;or\;E_{el\left(x,y,z\right)}-30\right)$$

The remaining descriptors are subjected to the ordered predictor selection (OPS), rearranged according to the informative contribution and auto-scaled as well (mean-centered and scaled to unity variance) prior to structure-activity model generation with the partial least squares (PLS) [69]. In order to investigate the QSAR model robustness the extensive validation is strongly advisable using the internal leave-several-out crossvalidation (LSO-CV) or activity-randomization (Y-scrambling) and external training/test set population analysis [70]. The model reliability and its applicability domain (AD) can be verified with Golbraikh & Tropsha criterion and leverage approach, respectively [71].

Finally, the interpretable spatial distribution of interactions can be plotted as three-dimensional color-coded contour maps (3D-pharmacophore) indicating the areas, where steric hindrance and/or charged substituents increase or demolish the binding affinity. Obviously, the incorporation of the target geometry into the LQTA protocol enables unbiased ligand alignment in the receptor-based superimposition as well as the induced fit simulation by exploration of mutual host-guest flexibilities [72]. Moreover, the Python programming language and Django framework were employed to implement a web-based and user-friendly graphical interface (called Web-4D-QSAR) integrating together the MDs and LQTAgrid modules [73].

The computational details of lattice-based 4D-QSAR approach were presented for a set of 4,5-dihydroxypyrimidine carboxamide derivatives acting as HIV-1 integrase (HIV-1 IN) inhibitors, benzo[e]pyrimido[5,4-b][1,4]diazepin-6(11*H*)-one derivatives as Aurora A kinase inhibitors, glycogen phosphorylase b inhibitors, MAP p38 kinase inhibitors, β-diketoacid (DKA) derivatives, dipeptidyl peptidase-IV (DPP-IV) inhibitors or trypanothione reductase inhibitors. In order to introduce the potential of the receptor-dependent LQTA-QSAR approach, an ensemble of phenothiazine derivatives that are specific competitive *Trypanosoma cruzi* trypanothione reductase (TR) inhibitors were investigated [66]. The binding mode of the phenotiazine analogues was evaluated in a simulated induced fit approach. The ligands’ alignments were explored based upon both ligand and binding site atoms, which is capable of providing unbiased CEP alignment. Based on the generated models, the binding mode of the bent tricyclic inhibitors of TR was postulated as well.

### 2.4. SiRMS 4D-QSAR Protocol

A novel 4D-QSAR approach based on the simplex representation of molecular structures (SiRMS) has been implemented in order to overcome the superimposition ambiguity for non-congeneric (heterogeneous) set of compounds—in this case the topological representation is not a limitation [74]. Structural topography (topology and stereochemical configuration) is encoded by a system of different simplexes defined as (un)bounded ‘tetraatomic fragments of fixed constitution, chirality and symmetry’ [75]. The overall number m of all available simplexes for *M*-atomic structure is specified according to $m=M!/\left(M-4\right)!\times 4!$ formula. Generally, simplexes are ranked hierarchically (1D→4D), where: 1D level is a combination of four atoms in the molecule; 2D level considers atomic connectivity, atom type and bond nature; 3D lever regards the molecular stereochemistry with chirality and symmetry; 4D level takes into account the probability of the particular conformer realization in the set of conformers [76]. Each structural parameter $S_{i}$ in 4D-QSAR modeling is computed by summing products of descriptors for each conformer $S_{k}^{i}$ and the probability of corresponding conformer $P_{k}$ as follows:

(5) $$S_{i}=\overset{N}{\underset{k=1}{\sum }}S_{k}^{i}\times P_{k}$$

where *N* is the number of conformers under consideration, $\;S_{k}^{i}$ is the *i*-th simplex descriptor value for conformer *k*. The probability of conformer realization $\;P_{k}$ is defined by its energy according to following formula:

(6) $$\;P_{k}={\left\{1+\overset{N}{\underset{i\neq k}{\sum }}\exp\left(\frac{-\left(E_{i}-E_{k}\right)}{RT}\right)\right\}}^{-1}$$

where $E_{i}$ and $E_{k}$ are energies of conformations *i* and *k*, respectively.

**Figure 7:**
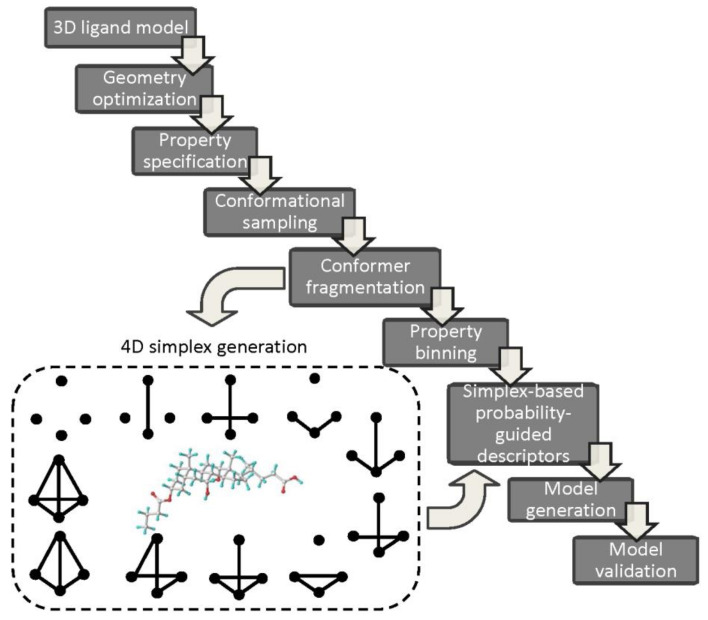
SiRMS protocol cascade.

The simplex-based 4D-QSAR operational cascade is briefly illustrated in Figure 7.

Typically, the generated, energetically-minimized 3D ligand models with the specified properties (e.g., charges, lipophilicities) are used to sample the conformational space of molecules (fourth dimension). Then, each conformer is fragmented into simplexes taking into account the individual atomic characteristics, for instance, atom type, partial charges, lipophilicity, refraction, electronegativity, hydrogen-bond nature (donor/acceptor), attraction or repulsion potentials, etc. [77]. The stereochemical configuration of simplexes is specified according to the modified Cahn-Ingold-Prelog (CIP) rules. Moreover, atoms can be divided into definite discrete subgroups correspondingly to some arbitrary formed ‘bucket’ boundaries for the partial charges or lipophilic values. The property-related simplex-retrieved representation of the conformational space for the ensemble of molecules arranged in the array (independent variables) and biological activities (dependent parameter) are then subjected to PLS analysis in order to establish QSAR relationship. The automatic variable selection procedures based on iterative (e.g., stepwise) or evolutionary (e.g., genetic) algorithms can be employed [78]. Obviously, the final verification of QSAR models using internal/external validation principles is a compulsory stage. Interestingly, a novel simplex-related determination of the applicability domain has been proposed with a vector that unifies two extreme points (active and inactive etalons) of the structural/property space depicting the directional changes (from the inactive to the active one) of toxicity in the variable space [79]. In order to simplify the interpretation of 4D simplex-based models the individual atomic contribution $C_{j}$ (positive or negative) can be color-coded according to the accumulation of regression coefficients $b_{j}$ for all investigated atom-containing simplexes M divided by the number of atoms in the particular simplex: $C_{j}=1/4\sum_{i=1}^{M}b_{i}$ [80]. In other words, the atoms or even molecular fragments that promote or interfere the modeled activity can be identified as well as the postulated ‘productive’ (active) conformations can be transferred to the subsequent 3D-QSAR analyses, respectively.

On the whole, the absence of molecular superimposition, diverse variants of atom differentiation and the explicit consideration of the stereochemical features in the conformational space are the practical pros of SiRMS 4D-QSAR protocol.

A multileveled system of the simplex representation of molecular structure was implemented in modeling of structure–anticancer/antiviral activity relationships for macrocyclic pyridinophanes, an affinity analysis of substituted piperazines, anticancer activity of macrocyclic pyridinophane derivatives or cytotoxicity and antiherpetic activity of N,N’-(bis-5-nitropyrimidyl)dispirotripiperazine derivatives. The operational details of simplex-based protocol were comprehensively presented by the assessment of the substitution characteristics of nitroaromatic compounds on the toxicity variations [80]. It was found that an aromatic ring with nitro group(s) contributes positively to toxicity, even though this contribution varies widely depending on the nature and number of other substituents. In most cases, insertion of fluorine and hydroxyl groups into nitroaromatics increases toxicity, whereas insertion of a methyl group has the opposite effect. Finally, some ‘toxicophore’ motifs were proposed using the hierarchical SiRMS protocol.

### 2.5. Hybrid 4D-QSAR Approach

**Figure 8:**
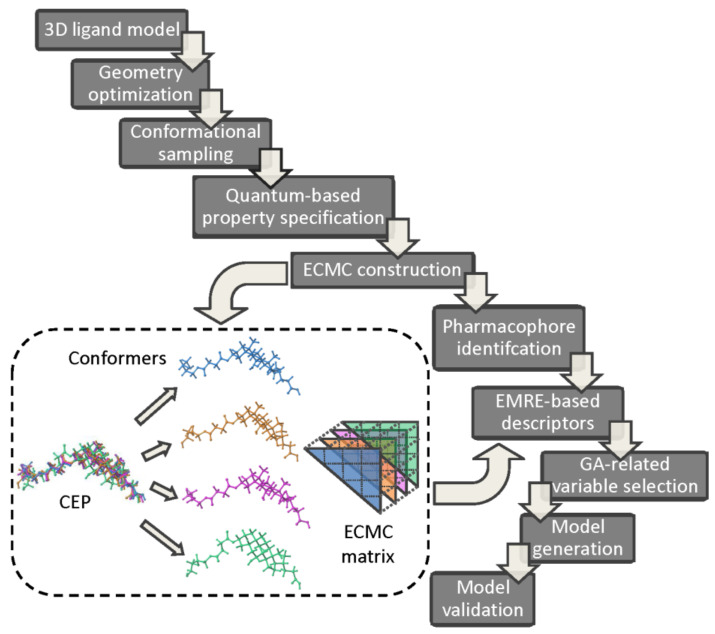
EC-GA 4D-QSAR operational workflow.

The pharmacophore identification and bioactivity prediction using the electron conformational-genetic algorithm (EC-GA) has been recently implemented in 4D-QSAR strategy in order to detect the impact of stereoisomerism on the variations in the biological responses [81]. A sophisticated hybrid combination of EC and GA rules incorporates conformational and superimposition freedom into the receptor-independent 4D-QSAR protocol. The electronic and geometric/topological characteristics of *n* atoms are arranged in the electron-conformational triangular matrix of congruity (ECMC) with *n(*n+1)/2 elements constructed for each conformer. Comparing ECMS elements, a smaller number of electron conformational submatrix of activity (ECSA) is specified within the given tolerances (a minimum one) in order to reveal the common pharmacophore pattern. The genetic algorithm and non-linear least square regression (PLS) are engaged to produce and validate the final 4D-QSAR models. The basic operational steps of the combined EC-GA approach are presented in Figure 8. The ligand 3D structures are constructed and optimized using the semi-empirical (PM3) or quantum chemical calculations (HF/6-311 G^**^ level in the aqueous medium) [82,83,84]. In order to generate the conformational ensemble profile for the investigated series Monte Carlo (MC) randomized search simulations are conducted. Each Boltzmann weighted conformer (densely populated) is characterized by triangular ECMC matrix with the local atomic characteristic (e.g., partial charges, valence activities, polarizabilities) on diagonal elements and the electron density distribution in 3D-space for bonded (Wiberg’s index) or unbounded atoms (interatomic distances) as off-diagonal elements, respectively. The lowest energy conformer of the most active molecule is chosen as a reference compound and intercompared with the remaining ECMCs within a predefined tolerance range to distinguish active from inactive compounds, respectively. The resulting electron conformational submatrix of activity (ECSA) represents a specific arrangement of functional groups in the active compounds (the pharmacophore pattern), where $P_{\alpha }$ and $\alpha_{a}$ demonstrate the probability of the pharmacophore occurrence in active and low/non-active compounds, respectively [85,86]:

(7) $$P_{\alpha }=\left(n_{1}+1\right)/\left(n_{1}+n_{3}+2\right)$$

(8) $$\;\;\alpha_{a}=\left(n_{1}\times n_{4}-n_{2}\times n_{3}\right)/\sqrt{\left(m_{1}\times m_{2}\times m_{3}\times m_{4}\right)}$$

where $n_{1}$, $n_{2}$ refer to the number of highly active and $n_{3}$, $n_{4}$ low active compounds bearing and non-bearing pharmacophoric pattern; $m_{1}$ and $m_{2}$ specify the number of highly active and weakly active molecules, whereas $m_{3}=n_{1}+n_{3};\;m_{4}=n_{2}+n_{4}.$ Roughly speaking, $P_{\alpha }$ proves the possibility of the pharmacophore existence in the active compounds, while $\alpha_{a}$ reflects the deposit of both active/low active molecules.

Additionally, antipharmacophore shielding (APS) and auxiliary (AG) atoms/fragments contribution to biological activity (negative or positive) can be introduced by means of the geometrical, electronic and physicochemical parameters using the cumulative function S as follows:

(9) $$Sn_{i}=\overset{N}{\underset{j=1}{\sum }}\kappa_{j}a_{ni}^{j}$$

where $a_{ni}^{j}$ are the parameters presenting the *j*-th type of the property impact in the *i*-th conformation of the *n*-th molecule; *N* is the number of chosen parameters; constant $\kappa_{j}$ shows the relative weights of the parameters on the activity [87,88,89].

Boltzmann weighted allotment of the compound’s conformer population to its activity in a function of the molecular descriptors, energy and temperature is computed according to the following formula:

(10) $$A_{n}=A_{l}\frac{\sum_{i=1}^{m_{l}}e^{-E_{li}/RT}\sum_{i=1}^{m_{n}}\delta_{ni}\left[Pha\right]e^{-S_{ni}}e^{-E_{ni/RT}}}{\sum_{i=1}^{m_{n}}e^{-E_{ni}/RT}\sum_{i=1}^{m_{l}}\delta_{li}\left[Pha\right]e^{-S_{li}}e^{-E_{li/RT}}}$$

where *δ* is a Kronecker delta function of two variables:

$$\delta_{ni}=\left\{\begin{array}{c} 0,\;Pha\;is\;absent\;\\ 1,\;Pha\;is\;present \end{array}\right.$$

and $A_{n}$ or $A_{l}$ correspond to activities of n-th and reference molecule l; $m_{n}$ or $m_{l}$ is the number of conformations of n-th and reference molecule l; $E_{li}$ or $E_{ni}$ represent the relative energy of i-th conformation of n-th and reference molecule l; R is gas constant; T is temperature. Since the activity A is exponentially depended on $S\left(A\sim e^{-S}\right)$ the quantitative A approximation is a function of the $a_{ni}^{j}$ selection and the corresponding parameter weight calculation (constant $\kappa_{j})$. The adjustable parameter $\kappa_{j}$ is mathematically optimized using a least square minimization of $\sum_{n}|A_{n}^{calc}-A_{n}^{exp}{|}^{2}$ as a function of constant $\;\kappa_{j}$ with the computed and experimental activities specified for the training set.

Subsequently, the EMRE-retrieved pool of descriptors including the topological, spatial or thermodynamic parameters is reduced using the stochastic, iterative and evolutionary GA procedure of the selection→mutation→reproduction→fitness assessment to eliminate uninformative variables [90,91,92]. Obviously, the selection of pertinent parameters (optimization) is a challenging issue of the structure-activity modeling process. Finally, the performance (robustness and predictive power) of the generated EC-GA models should be verified internally/externally using the training/test subset populations as recommended by the Organization for Economic Co-operation and Development (OECD) principles and Concordance Correlation Coefficient (CCC) criteria [93,94,95].

According to the available simulation data the EC-GA method seems to be a promising/effective tool for the comprehensive pharmacophore identification, relevant descriptor specification and activity calculation, respectively [96]. In fact, the hybrid 4D-QSAR approach was successfully employed for pharmacophore identification of pyrazole pyridine carboxylic acid derivatives, pyrrolo[2,1-c][1,4]benzodiazepines, N-morpholino triaminotriazine derivatives, penicillins, 1,4-dihydropyridines, benzotriazines, HEPT analogues or tetrahydroimidazo[4,5,1-jk][1,4]benzodiazepinone (TIBO) derivatives. The ‘synergetic’ effect of the EC-GA conjugation (pharmacophore identification and bioactivity prediction) was presented in modeling of antibacterial activity for the set of β-lactam antibiotics (known as penicillin) [81]. According to the generated 4D-QSAR models with good descriptive and predictive performance a new model was constructed and optimized using only one conformer of each compound(3D-QSAR). It was also postulated that penicillin activity was controlled by a pharmacophore containing seven atoms with certain electronic and geometrical characteristics.

### 2.6. Quasar 4D-QSAR Approach

**Figure 9:**
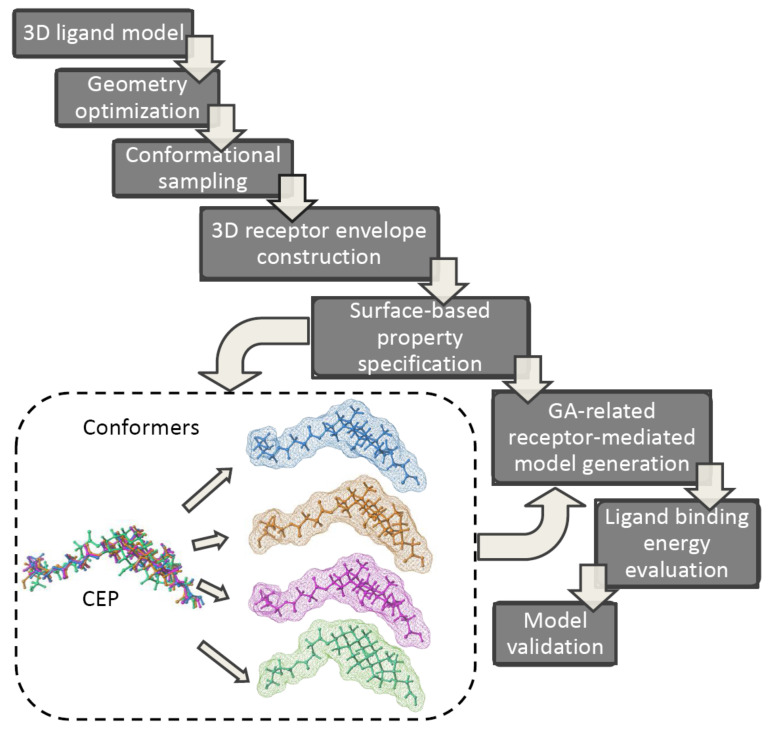
Quasi 4D-QSAR cascade.

In order to address the shape-dependent complementarity pitfall at the stage of the host-guest complex formation (e.g., the induced fit or H-bond flip-flop) the quasi-atomistic receptor surface modeling approach (Quasar) has been proposed in the multidimensional-QSAR as a conceptual ‘bridge’ between the RI and RD protocols, respectively [97]. The manifestation/magnitude of the local induced fit (the ligand-mediated adaptation of binding pocket to molecular topology/topography) or simulation of the H-bond flip-flop particles (Ser, Thr, Tyr, Cys, His, Asn or Gln amino-acids acting simultaneously as HB donors/acceptors due to a conformationally flexible H-bonding functions) are still challenging issues in the rational drug design. In consequence, the multiple ligand conformation, orientation and protonation representation has been enhanced by an additional level of model abstraction (degree of freedom)—the topology of the quasi-atomistic receptor surrogate [98,99]. The conceptual cascade of the Quasar 4D-QSAR is presented in Figure 9. An averaged peptidic pseudoreceptor-surface family is constructed as a 3D ‘inner’ envelope randomly populated with atomistic properties (e.g., hydrophobicity, partial charge, electrostatic potential, H-bonding propensity), that surrounds the ligands of the training set at the Van der Waals distance (radius 0.8 Å) [100]. Obviously, the hypothetical shape of the receptor surface mirrors vaguely the steric nature of the binding site. In practice, the ligand conformational space within this primordial envelope can be scanned with Monte Carlo (MC) search algorithm. Next, the evolution of the initial family of receptor models is performed by means of the genetic operators (crossover and random mutation) and the estimation of relative free energies of ligand binding towards pseudoreceptor models is conducted as well. A normalized Boltzmann distribution is used to evaluate the conformer energy contribution to the total energy [101,102]. Finally, the scrambling tests are employed to validate a family of receptor models using the training/test subsets of molecules. Practically, the binding data of training population are arbitrarily and recurrently scrambled with respect to the true activities. Hence, based on the scramble-derived model the binding energies for test set are foreseen—the higher prediction accuracy the worse model is due to insensitivity towards the biological data [103].

The multiple-conformational (4D) Quasar approach provides an elegant way to estimate the receptor-mediated interaction energies by populating receptor surface models with atomic properties. In fact, the methodology allows for a subtle scaling of energetic contributions of geometrically flexible molecules reducing the alignment bias. The adjustment of an ‘averaged’ pseudoreceptor to an individual ligand geometry and the conformational mobility of H-bond functionality can be simulated as well [104].

In order to constrain the superimposition bias in multi-conformational ligand representation the Quasar concept was engaged to establish QSARs for neurokinin-1 receptor antagonists and aryl hydrocarbon receptor antagonists (dibenzo-dioxins, dibenzofurans, biphenyls, and polyaromatic hydrocarbons), dopamine β-hydroxylase inhibitors and aryl hydrocarbon receptor antagonists or 5-HT_2A_ receptor antagonists, respectively. Moreover, an automated quasi-4D-QSAR that mimicked the multi-way-PLS analyses to provide predictive SAR models for highly flexible CXCR4 cyclic pentapeptide inhibitors was proposed [97]. The bioactive conformer geometry and superimposition rule were specified in the recurring loop of the activity-descriptor regression examination for the conformer ensembles using two-way PLS protocol. The side chains of the cyclic pentapeptides were indicated as the functional groups interacting with the CXCR4 receptor.

### 2.7. 4D-QSAR: Happy Stories

The detailed description of successful applications of RI/RD-4D-QSAR paradigm is beyond the scope of this paper and it can be found elsewhere [105,106,107,108,109,110,111,112,113,114,115]. The rough characterization of the scientific projects, where 4D-QSAR methodology was implemented with the specification of the applied protocol, objects of interests and references is reported in Table 1.

## 3. 4D-QSAR: Twilight or Bright Future Perspective?

In summary, an outline of the current state-of-the-art in the area of ligand-based and receptor-mediated 4D-QSAR is provided. The idea underlying 4D-QSAR assumes that a molecule represented by several conformers is regarded as a particular case of the multiple instance learning modeling—the Boltzmann average spatial distribution of the molecular shape is a function of biological response variations. Obviously, the conformation-related characteristics of the molecular flexibility and dynamic interactions with the target cannot be captured by models trained on the chirality-unaware descriptors that are regarded as a ‘bottleneck’ of the single-conformer 3D procedures. As a matter of fact, 4D-QSAR approach has been experiencing a promising renewal of interest that might be fueled by the rising power of the graphics processing unit (GPU) clusters applied to the full-atom MD-based simulations of protein-ligand complexes. For instance, Compute Unified Device Architecture (CUDA) is a promising computing technology useful for support of general purpose and parallel processing on graphics accelerators. Simply, the more computational power the longer host-guest dynamic description (molecular trajectory). In practice, a range of 4D-QSAR procedures (namely dialects) have been employed for various series of compounds. On the other hand, new types of chirality-aware descriptors to encode the conformational diversity, especially in the context of the multifaceted ligand-protein (non-)covalent forces, are urgently needed. It seems, that after the years of stagnation in 4D-QSAR development associated with CPU-limited emulation of the host-guest interactions, the method has a brighter future ahead.

To the best of my knowledge, there is no other review that gathers the ‘mainstream’ algorithms of 4D-QSAR together.

## Figures and Tables

**Table 1:** Brief characterization of 4D-QSAR protocols, research projects with references.

Methodology	Protocol	Research Subject	References
Hopfinger’s 4D-QSAR	RD	4-hydroxy-5,6-dihydropyrone analogues as HIV-1 protease inhibitors	Santos-Filho, O.A. et al. [29]
	RI	norstatine derived inhibitors of HIV-1 protease based on the 3(S)-amino-2(S)-hydroxyl- 4-phenylbutanoic acid core (AHPBA)	Senese, C.L. et al. [28]
	RD	glucose inhibitors of glycogen phosphorylase b, GPb.	Pan, D. et al. [30]
	RD	pyridinyl-imidazole and pyrimidinylimidazole inhibitors of p38-mitogen-activated protein kinase (p38-MAPK)	Romeiro, N.C. et al. [32]
	RD	C2-symmetric diol inhibitors of HIV-1 protease(HOE/BAY-793 analogues)	da Cunha, E.F.F. et al. [35]
	RD	2-arylbenzothiophene derivatives	Sodero, A.C.R. et al. [58]
	RD	glucose analogue inhibitors of glycogen phosphorylase (GPb)	Pan, D. et al. [44]
	RD	peptides reversible inhibitors of Trypanosoma cruzi trypanothione reductase (TR)	Silva da Rocha Pita, S. et al. [56]
	RD	β-N-biaryl ether sulfonamide hydroxamate derivatives as potent inhibitors against matrix metalloproteinase subtype 9 (MMP-9)	Turra, K.M. et al. [88]
	RI	hydrazides	Pasqualoto, K.F.M. et al. [34]
	RI	lamellarins against human hormone dependent T47D breast cancer cells	Thipnate, P. et al. [33]
	RI	5′-thiourea-substituted R-thymidine inhibitors	Andrade, C.H. et al. [26]
	RI	7-oxabicyclo[2.2.1]heptane oxazole thromboxane A_2_ (TXA_2_) receptor antagonists	Albuquerque, M.G. et al. [43]
	RI	antiarrhythmics agents	Klein, C.D.P. et al. [55]
	RI	propofol (2,6-diisopropylphenol) analogues	Krasowski, M.D. et al. [45]
	RI	benzothiophene analogs as dopamine D2 receptor inhibitors.	Caldas, G.B. et al. [72]
	RI	tetrahydropyrimidine-2-one based inhibitors of HIV-1 protease	Senese, C.L. et al. [28]
	RI	azole antifungal P450 analogue inhibitors	Liu, J. et al. [47]
	RI	glucose inhibitors of GPb.	Hopfinger, A.J. et al. [11]
	RI	antifolates and pyrrolo[2,3-d]pyrimidines as antimalarial dihydrofolate reductase inhibitors	Santos-Filho, O.A. et al. [49]
	RI	benzylpyrimidine inhibitors of dihydrofolate reductase, prostaglandin PGF_2_α antinidatory analogs, dipyridodiazepinone inhibitors of HIV-1 reverse transcriptase	Hopfinger, A.J. et al. [1]
	RI	glucose analog inhibitors of glycogen phosphorylase	Venkatarangan, P. et al. [40]
	RI	flavonoids	Hong, X. et al. [36]
	RI	ecdysteroids	Ravi, M. et al. [50]
	RI	thymidine-based inhibitors of monophosphate kinase (TMPK) as potential antituberculosis agents	Andrade, C.H. et al. [25]
	RI	Leishmania donovani *N*-myristoyltransferase (NMT) inhibitors	Santos-Garcia, L. et al. [112]
	RI	glucose analogue inhibitors of glycogen phosphorylase	Hopfinger, A.J. et al. [40]
	RI	ecdysteroids and diacylhydrazines	Hormann, R.E. et al. [105]
SOM 4D-QSAR	RD	anthraquinone dyes	Bak, A. et al. [62]
	RI	benzoic acids, azo dyes, and steroids	Bak, A. et al. [59]
	RI	benzoic acids	Polanski, J. et al. [6]
	RI	1-[2-Hydroxyethoxy) methyl]-6-(phenylthio)-thymines (HEPT)	Bak, A. et al. [60]
	RI	2,4-diamino-5-benzylpyrimidine inhibitors	Polanski, J. et al. [61]
	RI	cholic acid derivatives	Bak, A. et al. [63]
LQTA 4D-QSAR	RI	3-pyrazolyl substituted coumarin derivatives	Patil, R. et al. [70]
	RD	phenothiazine derivatives as trypanothione reductase inhibitors	Barbosa, E.G. et al. [66]
	RD	Gram-negative specific LpxC inhibitors	Ghasemi, J.B. et al. [68]
	RI	glycogen phosphorylase b inhibitors and MAP p38 kinase inhibitors	Martins, J.P.A. et al. [64]
	RI	Β-diketo acid derivatives as HIV-1 IN strand transfer inhibitors (INSTI)	de Melo, E.B. et al. [65]
	RI	benzo[*e*]pyrimido[5,4-*b*][1,4]diazepin-6(11*H)*-one as as Aurora A kinase inhibitors	Kanhed, A.M. et al.[67]
	RI	4,5-dihydroxypyrimidine carboxamide derivatives	Martins, J.P.A. et al. [73]
Simplex 4D-QSAR	RI	macrocyclic pyridinophane analogues	Kuzmin, V.E. et al. [74]
	RI	substituted piperazines	Kuzmin, V.E. et al. [77]
	RI	macrocyclic pyridinophane analogues	Kuzmin, V.E. et al. [75]
	RI RI RI	[(biphenyloxy)propyl]isoxazole derivatives nitroaromative derivatives	Kuzmin, V.E. et al. [78] Kuzmin, V.E. et al. [79]
Quasi 4D-QSAR	RI	neurokinin-1 receptor antagonists	Vedani, A. et al. [104]
	RI	neurokinin-1 receptor antagonists and aryl hydrocarbon receptor antagonists (dibenzodioxins, dibenzofurans, biphenyls, and polyaromatic hydrocarbons)	Vedani, A. et al. [103]
	RI	dopamine β-hydroxylase inhibitors and aryl hydrocarbon receptor antagonists	Vedani, A. et al. [102]
	RI	phenylalkylamines, tryptamines, ergolines as 5-HT_2A_ receptor antagonists	Streich, D. et al. [99]
	RI	CXCR4 cyclic pentapeptide inhibitors	Bhonsle, J.B. et al. [97]
Hybrid 4D-QSAR	RI	penicillin analogues	Yanmaz, E. et al. [81]
	RI	tetrahydroimidazo[4,5,1-jk][1,4]benzodiazepinone (TIBO) derivatives	Akyüz, L. et al. [82]
	RI	1-[(2-hydroxyethoxy)-methyl]-6-(phenylthio) thymine (HEPT) derivatives	Akyüz, L. et al. [83]
	RI	benzotriazine derivativesas as sarcoma inhibitors	Sahin, K. et al. [85]
	RI	*N*-morpholino triaminotriazine derivatives	Saripinar, E. et al. [86]
	RI	ruthenium(II) arene complex derivatives	Yavuz, S.C. et al. [87]
	RI	pyrrolo[2,1-c][1,4]benzodiazepine derivatives	Özalp, A. et al. [89]
	RI	pyrazole pyridine carboxylic acid derivatives	Tüzün, B. et al. [90]
	RI	alkynylphenoxyacetic acid analogues as CRTh2 (DP2) receptor antagonists	Köprü, S. et al. [91]
	RI	phosphoinositide-3-kinase (PI3K) inhibitors	Safavi-Sohi, R. et al. [98]
	RI	dipeptidyl boronic derivatives as proteasomeinhibitors	Catalkaya, S. et al. [95]

## Data Availability

Not applicable.
